# Supplementary material for: MLMarker: a machine learning framework for tissue inference and biomarker discovery
Source: Genome Biol. 2026 Jun 24;27:207. doi: 10.1186/s13059-026-04125-8 (PMC13292323; doi:10.1186/s13059-026-04125-8)
Supplement: Supplementary file 1 — Supplementary Material 1. [file 13059_2026_4125_MOESM1_ESM.docx]

## Supplementary materials


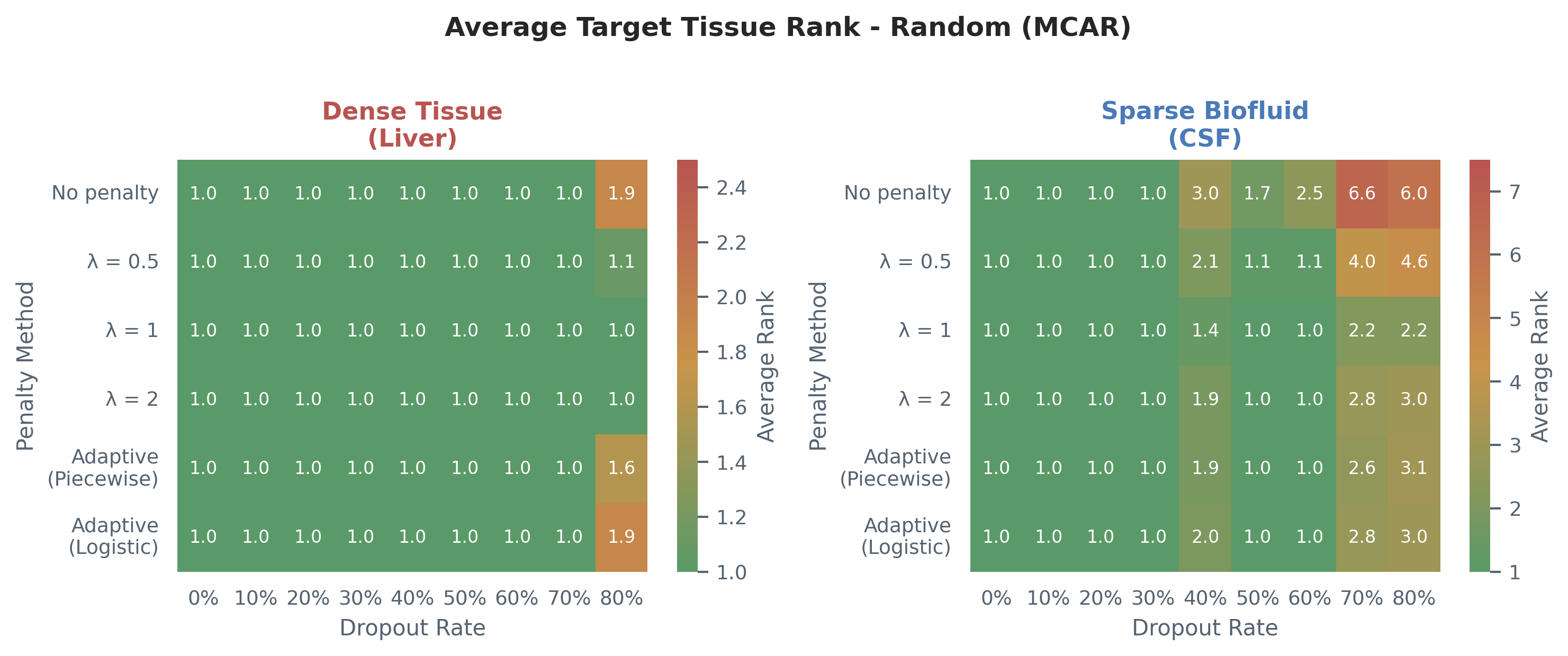


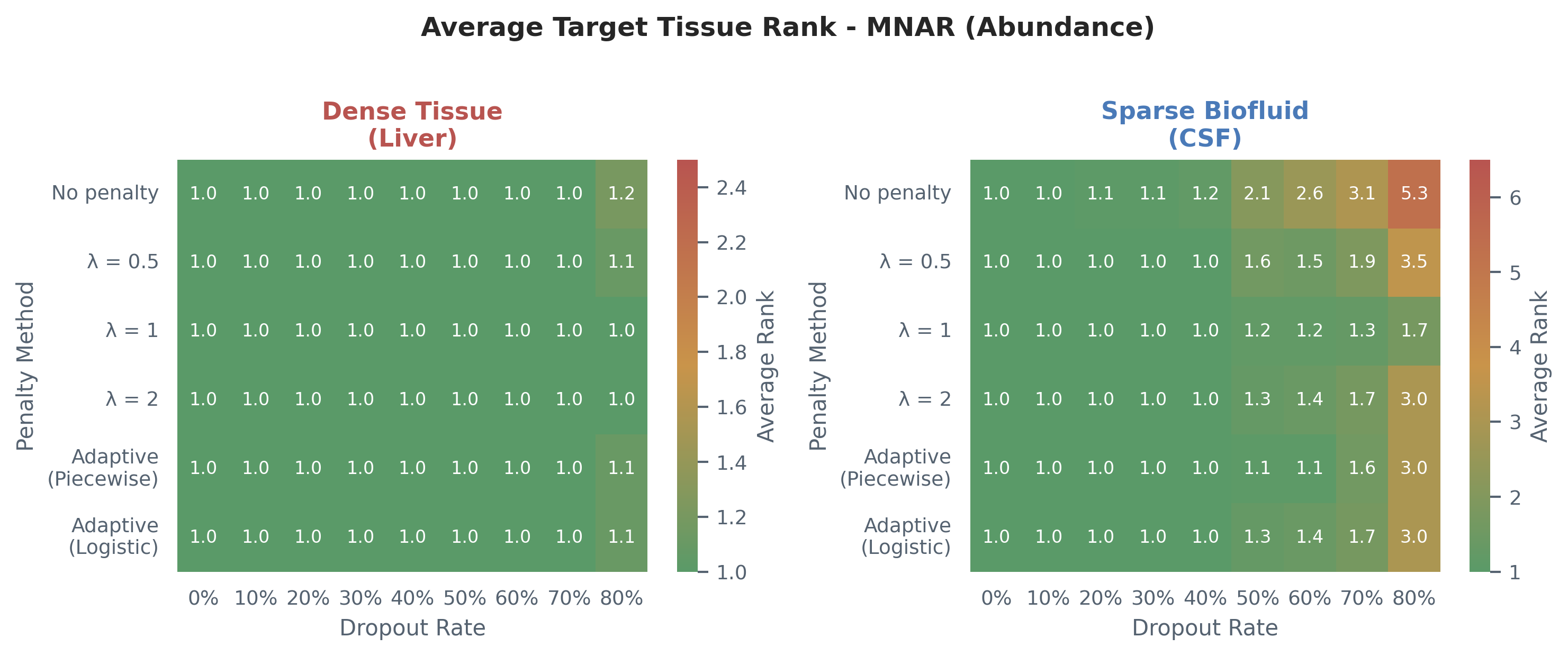


*Fig S1 Average rank degradation of the correct tissue prediction under MCAR (top) and MNAR (bottom) missingness across penalty strategies for dense tissue (liver, left) and sparse biofluid (CSF, right). Optimal performance is indicated by a limited rank degradation. Each heatmap displays the average rank of the correct tissue prediction across increasing dropout rates (x-axis, 0–80%) for six penalty methods (y-axis): no penalty, fixed λ values (0.5, 1, 2), and adaptive strategies (piecewise, logistic). In both samples, λ = 1 consistently maintains low rank values across all dropout levels, outperforming other methods especially under high missingness*


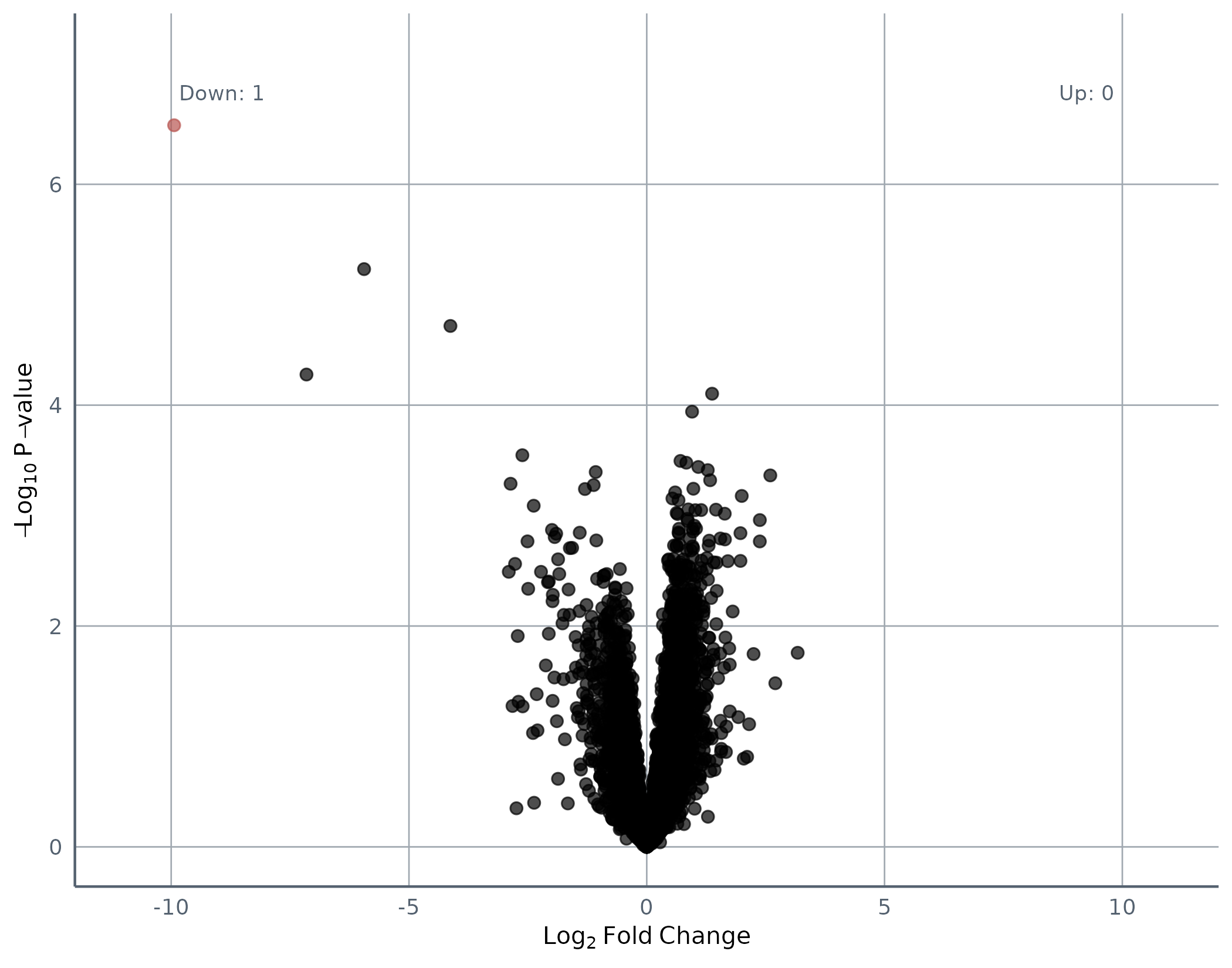


*Fig S2 Differential expression analysis volcano plots between group A and group B with log fold change on X-axis, and -log_10_ adjusted p-value following Benjamini-Hochberg correction on Y-axis. Red dots indicate proteins with adjusted p-value < 0.01. Groups are previously determined through hierarchical clustering. Only 1 significantly downregulated protein in group B could be identified.*


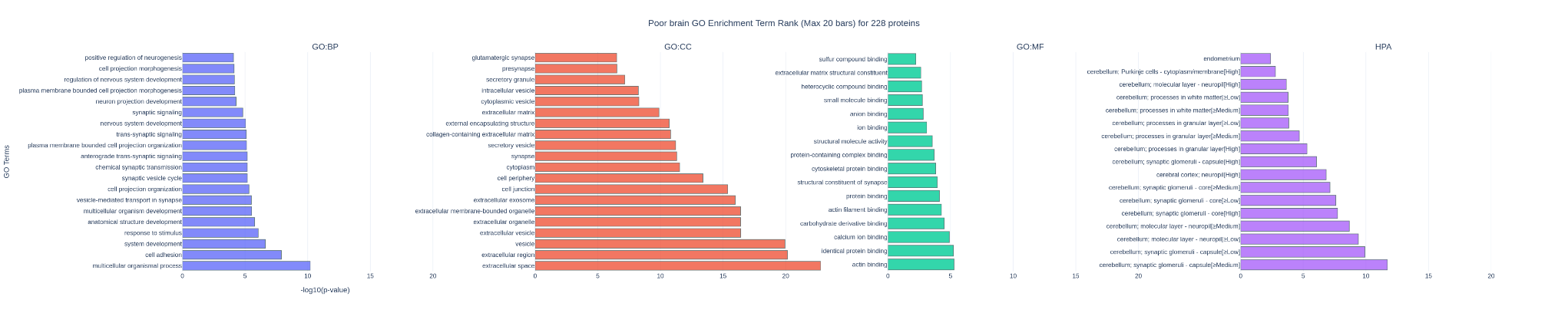

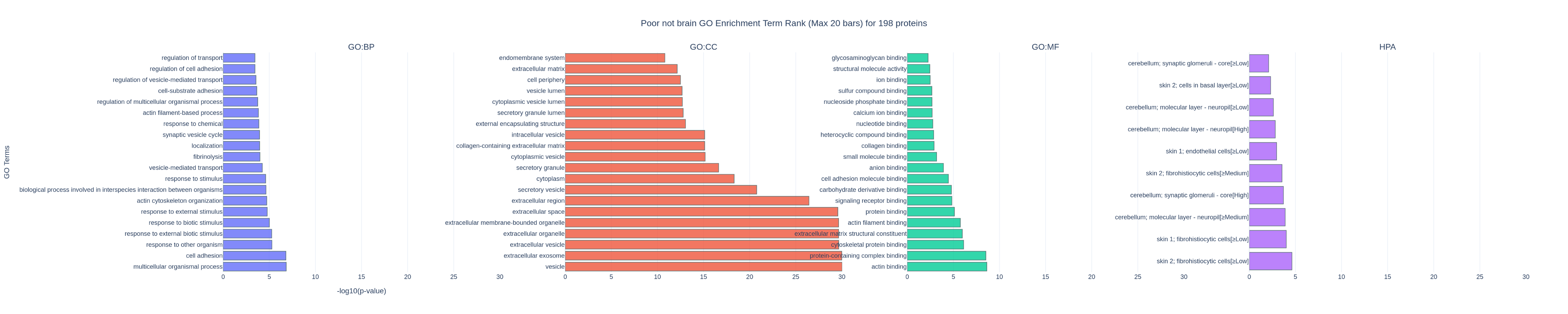

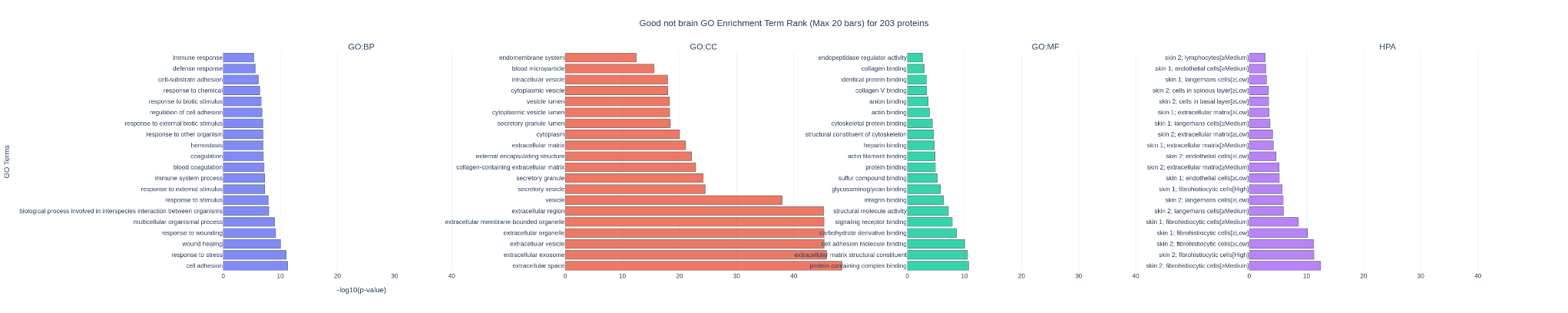

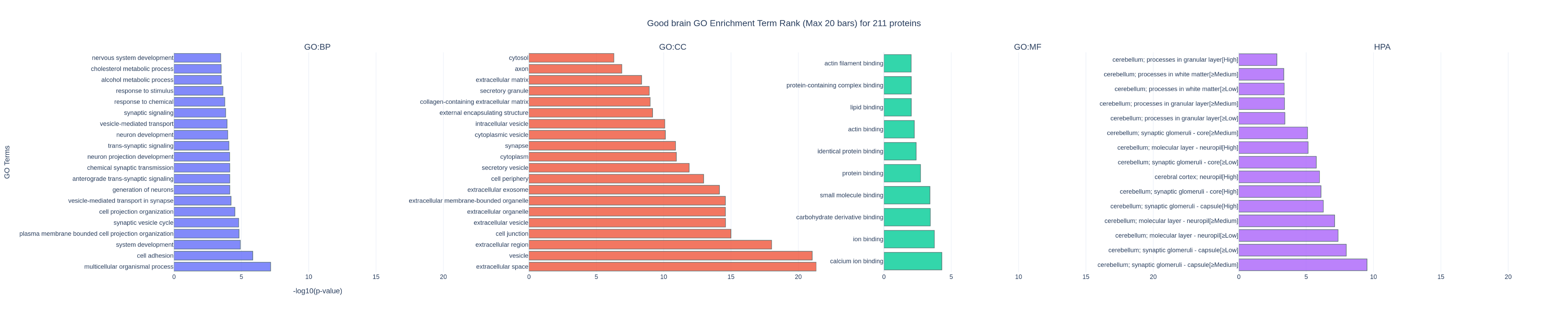


*Fig S3: Over-representation analysis of the top 1% highest ranking proteins for the four subpopulations at p-value of 1%.*


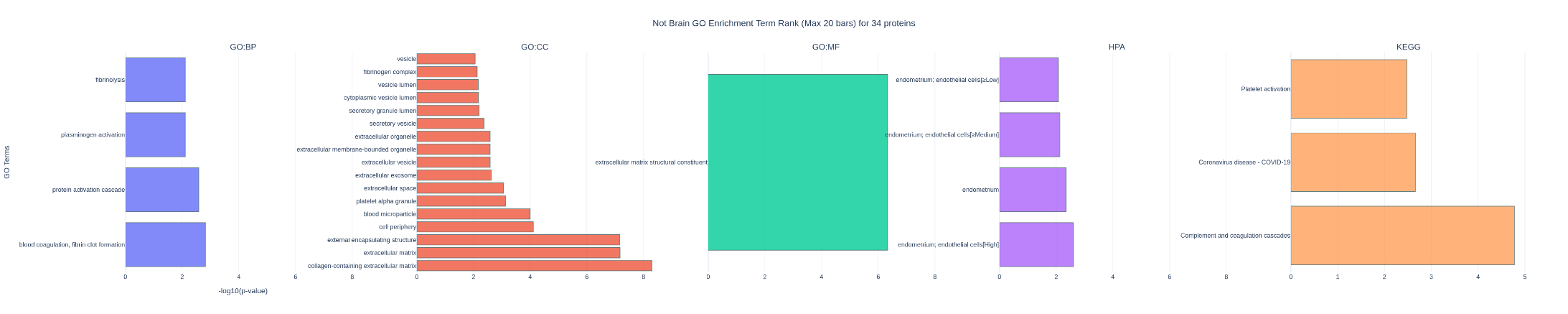

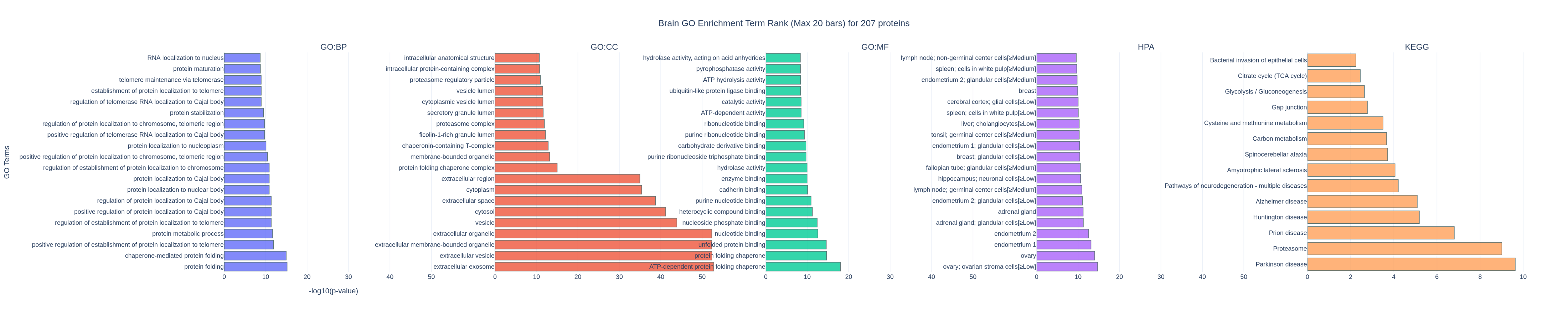


*Fig S4: Over-representation analysis of the 255 differentially expressed proteins upregulated in the not-brain predicted samples (top) and brain predicted samples (bottom).*

*
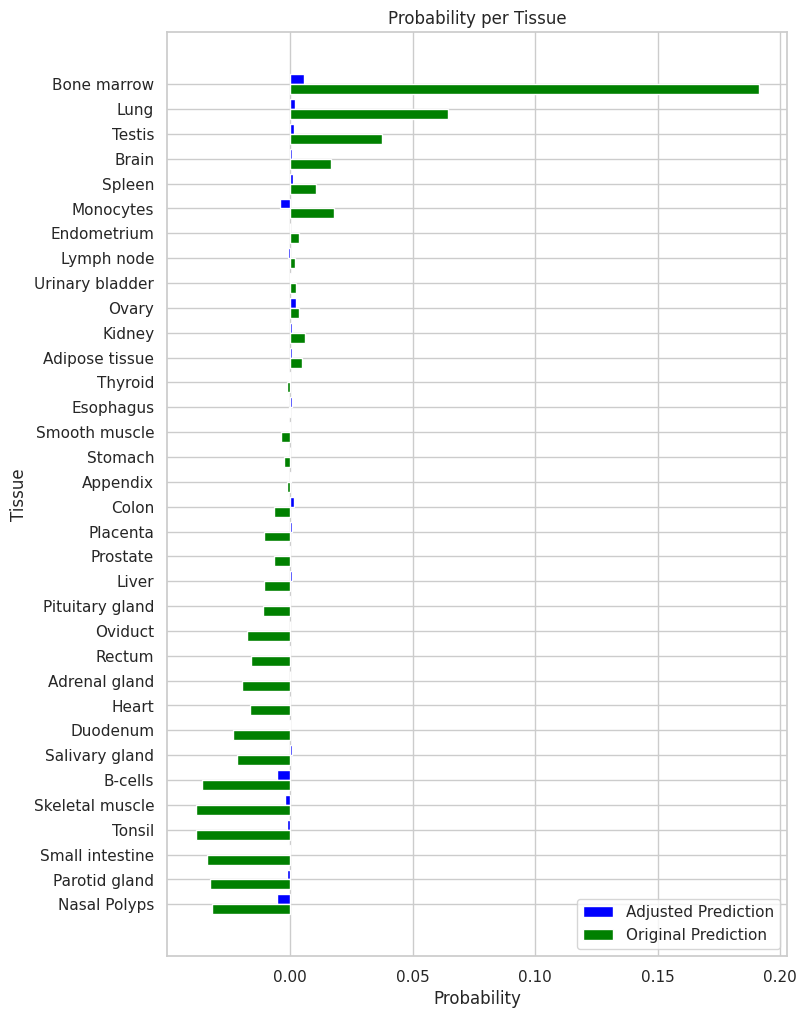
*

*Fig S5: Bar plot showing tissue similarity scores for a fully zeroed sample. Original predictions are shown in green; adjusted predictions using the penalty factor are shown in blue. The application of the penalty factor results in a strong reduction in prediction confidence across all tissues.*


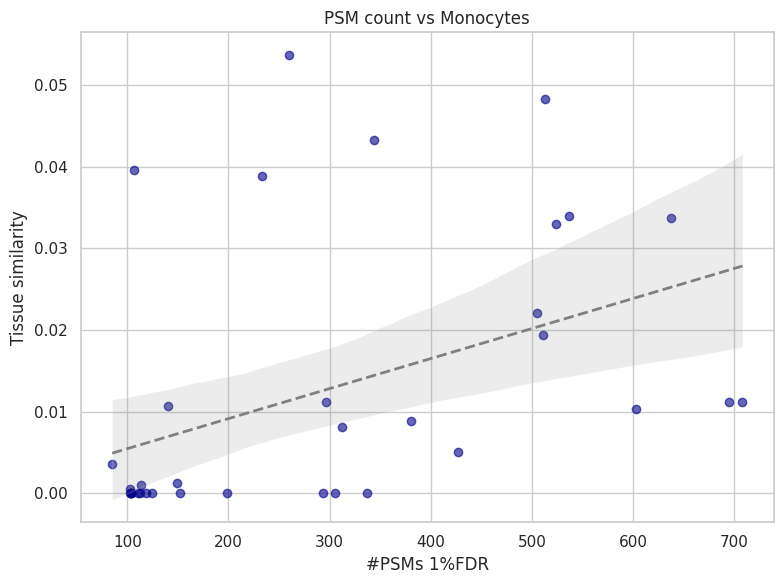

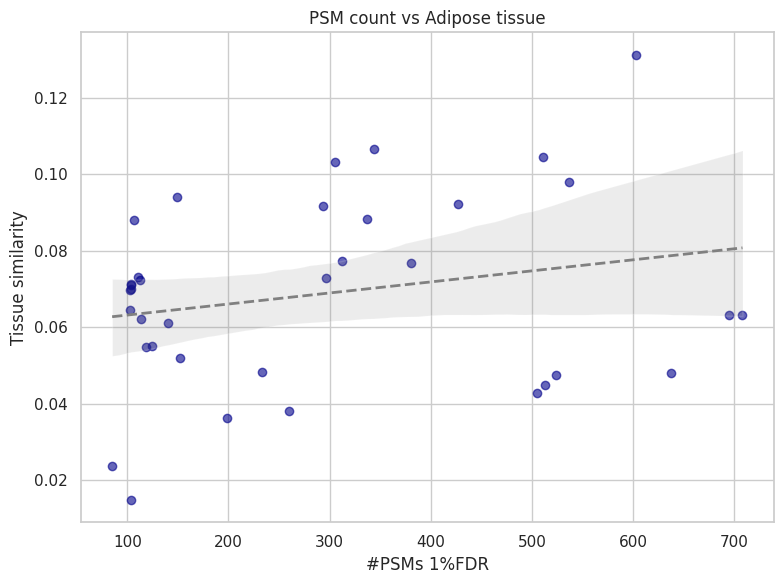

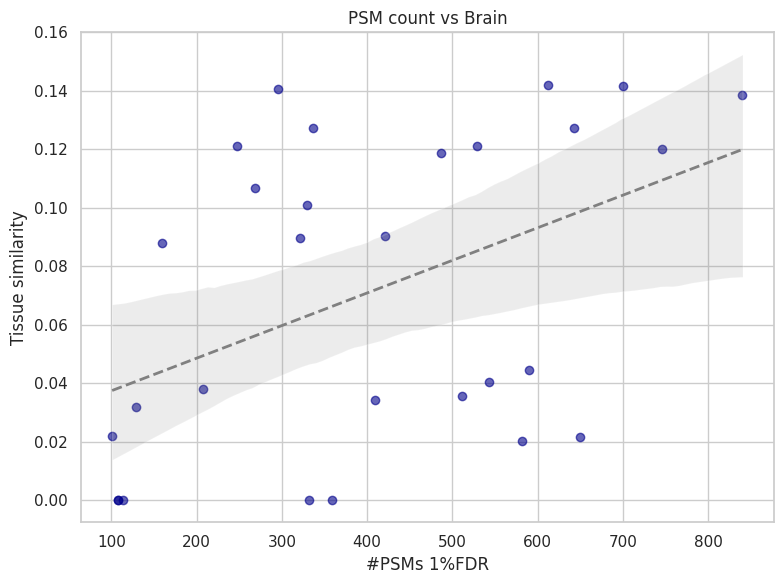

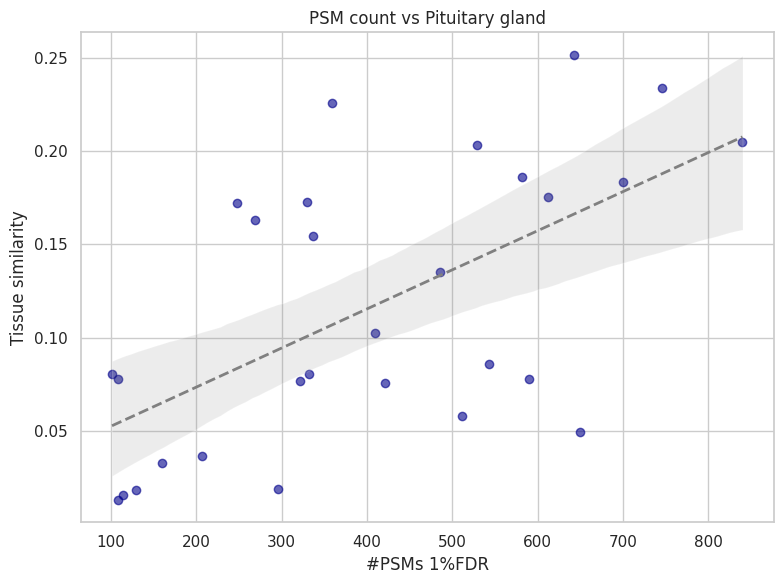


*Fig S6: Scatterplot showing the relationship between tissue similarity and PSM counts for pituitary gland (top left), brain in the CSF samples (top right) and for adipose tissue (bottom left) and monocytes in plasma (bottom right). The x-axis represents the number of PSMs passing the 1% FDR threshold. A linear regression trendline indicates that an increase in PSMs often leads to greater certainty in tissue similarity predictions.*

*
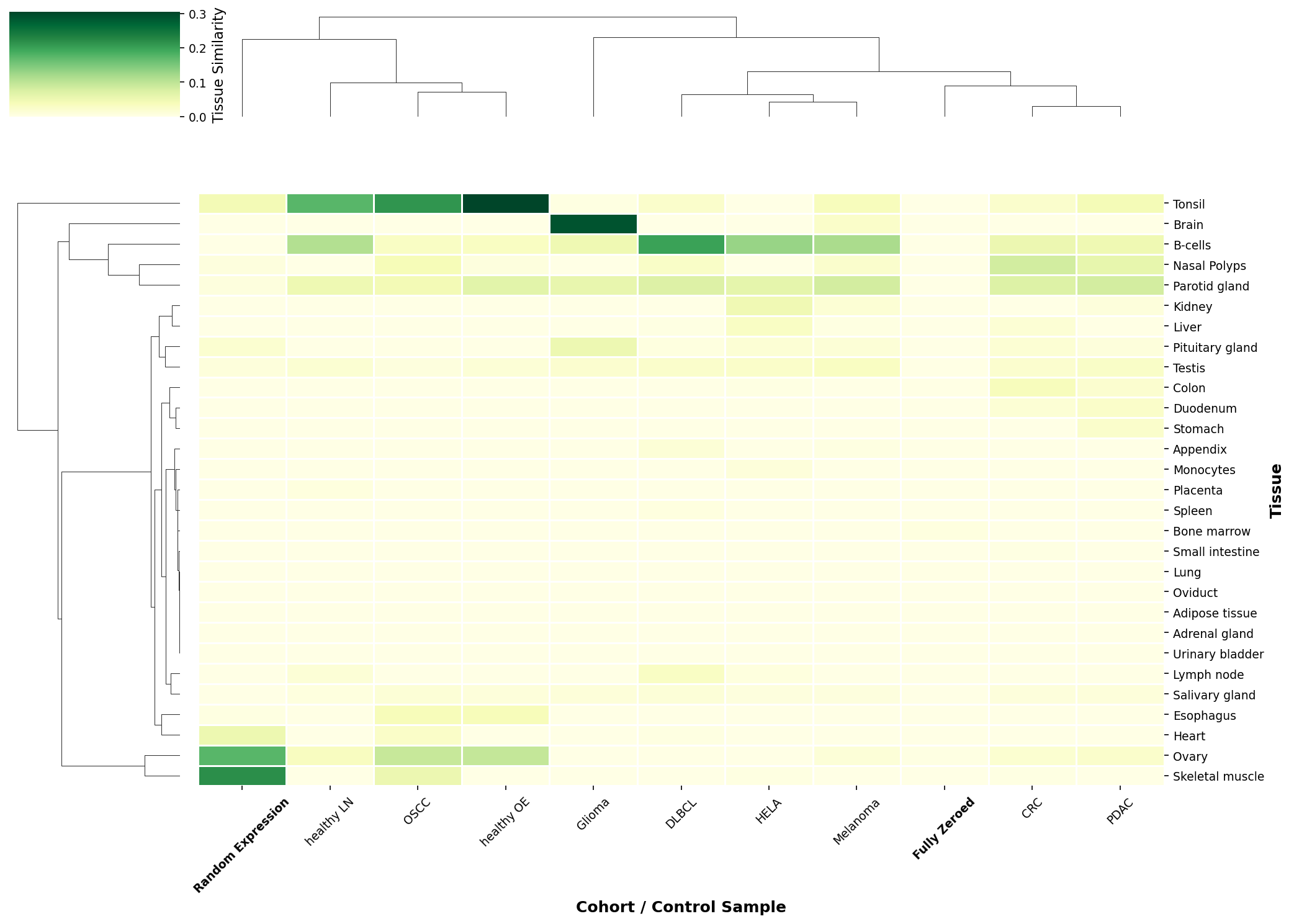
*

*Fig S7: Dendrogram showing tissue similarity per cancer type, including random and fully zeroed samples (highlighted)*
